# Characteristics, clinical and laboratory data and outcomes of pregnant women with confirmed SARS-CoV-2 infection admitted to Al-Zahra tertiary referral maternity center in Iran: a case series of 24 patients

**DOI:** 10.1186/s12884-021-03764-y

**Published:** 2021-05-17

**Authors:** Maryam Vaezi, Mojgan Mirghafourvand, Shahla Hemmatzadeh

**Affiliations:** 1 Department of Obstetrics and Gynecology, School of Medicine, Women’s Reproductive Health Research Center, Clinical Research Institute, Alzahra Hospital, Tabriz University of Medical sciences, Tabriz, Iran; 2 Department of Midwifery, Social Determinants of Health Research Center, Tabriz University of Medical Sciences, Tabriz, Iran; 3 Students’ Research Committee, Department of Midwifery, Faculty of Nursing and Midwifery, Tabriz University of Medical Sciences, Tabriz, Iran

**Keywords:** COVID-19, Iran, Pregnancy, Case series

## Abstract

**Background:**

Physiological changes during pregnancy put pregnant women at higher risk for COVID-19 complications. The objective of this study was to evaluate clinical and laboratory characteristics and outcomes of 24 COVID-19 pregnant patients and their newborns referred to the Al-Zahra tertiary maternity hospital in Tabriz, Iran.

**Methods:**

Clinical records of 24 COVID-19 confirmed pregnant patients were retrospectively reviewed from10 March 2020 to 15 April 2020. Vertical transition was assessed through neonatal pharyngeal swab samples. The study has been approved by the Tabriz University Medical Ethics Committee (IR.TBZMED.REC.1399.497).

**Results:**

There were 24 hospitalized cases with clinical symptoms and confirmed diagnosis of COVID-19. The mean age of cases was 26.5 years; most were nulliparous (54.2%), in their third trimester (62.5%) and were in the type A blood group. Clinical symptoms in order of prevalence were cough, fever, dyspnea, myalgia, anosmia, and diarrhea. Oxygen saturation (SpO2) in 70.8% cases was in the normal range (greater than 93%). The risk of premature labor or abortion in cases showed no increase. 12 cases were in ongoing normal status; on follow up, 11 cases had delivered their babies at term and one had ended in IUFD because of pregnancy-induced hypertension. All delivered babies were healthy. Caesarean section in all cases was performed under obstetric indications or maternal demand, and no relation was found between COVID-19 and Caesarean delivery. Neonatal outcomes according to gestational age in 8 cases out of 11 (72.72%) were desirable; neonatal morbidity and mortality resulted from pregnancy complications. Blood pH in 6 neonates was assessed due to immaturity and NICU admission, all of which were in normal ranges except one case related to HELLP syndrome. There was no evidence of vertical transmission.

**Conclusions:**

Findings suggest that clinical symptoms in pregnancy were similar to non-pregnant women, no rise in risk of premature labor or abortion was seen, and vertical transmission was not observed in none of cases. Lymphopenia was the leading laboratory change. Given asymptomatic cases despite severe forms of infection in pregnancies, we propose screening in all suspected cases. All placentas and newborns should be tested in the field for vertical transmission.

## Background

During the most recent year, in which COVID-19 (SARS-COV-2) has spread around the world, many studies have investigated pregnancy changes and outcomes. Most studies indicated that the risk of infection in pregnant women is not different from non-pregnant women [1, 2]. However, recent data from the Centers for Disease Control and Prevention (CDC) over a large pregnant population with confirmed COVID-19 suggest that pregnant women may have increased risk for severe illness from COVID-19 compared to non-pregnant women [3]. Previous studies have found that the severe form of the disease is more prevalent in the third trimester [4, 5].

COVID-19, like other types of coronaviruses, can cause complications especially in pregnant populations [6, 7]. Immunologic and physiologic changes in pregnancy may explain the higher risk of complications. Maternal and fetal complications include spontaneous miscarriage, premature labor, intra-uterine growth retardation (IUGR), tracheotomy and mechanical ventilation, admission to the intensive care unit, renal failure, and disseminated intravascular coagulation [8].

The COVID-19 virus has not yet been detected in breast milk. The primary route of neonatal infection is through respiratory contact with the mother or an infected member of the family [9]. One study recommended the newborns of suspected or confirmed COVID-19 mothers must be separated for two weeks, not be fed by the mother’s breast milk, and close contact with the mother should be prevented [10]; however, this recommendation is inconsistent with the advice given by WHO and UNICEF. Although currently there is no evidence for intrauterine infection in confirmed COVID-19 pregnant women in late pregnancy, the risk of fetal and neonatal infection is among the major concerns in pregnant woman [11].

Further studies are needed for the management of healthy pregnancies. The current study aimed to provide clinical and laboratory characteristics and outcomes of 24 COVID-19 pregnant patients and their newborns who were referred to the Al-Zahra tertiary maternal referral hospital in Tabriz, Iran.

## Methods

The records of all pregnant women with confirmed COVID-19 infections were assessed retrospectively from March 10 to April 15, 2020. The investigation was made according to clinical symptoms, blood group type, laboratory characteristics, pregnancy outcomes, and neonatal outcomes. Most of the women were referred with clinical symptoms. Comorbidities such as preeclampsia, diabetes mellitus or other diseases were assessed and documented. Some women were referred from the surrounding suburbs. The Al-Zahra hospital in Tabriz is a tertiary center for pregnant women in the northwest. In all, 24 files of hospitalized patient were assessed and the results were analyzed. Written informed consent was obtained from all pregnant women for publication of their data. The study was reviewed and approved by the Tabriz University Medical Ethics Committee with the code IR.TBZMED.REC.1399.497.

## Results

### Clinical and pregnancy characteristics

In total, 24 cases were hospitalized with confirmed COVID-19. The mean age was 26.5 years (range: 17–39), most cases were in their third trimester (15 cases, 62.5%), 6 cases were in their second trimester (25%), and only 3 cases (12.5%) were under 14 weeks. Thirteen cases were nulliparous (54.2%), 10 cases were in their second pregnancy (41.7%), and one of them was in their fifth pregnancy. Regarding their COVID-19 diagnosis, all patients were referred with clinical symptoms and their diagnosis was confirmed with a PCR test (in 21 cases) and CT scans (in 3 cases). Three cases were positive for both (a CT scan was performed with very low-dose radiation for respiratory symptoms according to the Iranian health ministry COVID-19 guidelines). Clinical symptoms are shown in Table 1. The most common symptoms were fever and cough.

**Table 1:** Clinical characteristics of 24 pregnant patients with COVID-19

Characteristics	Number (%)
Fever	14 (58.3)
Cough	15 (62.5)
Dyspnea	10 (41.7)
Myalgia	3 (12.5)
Diarrhea	1 (4.2)
Sore throat	1 (4.2)
Anosmia	1 (4.2)

**Fig. 1:**
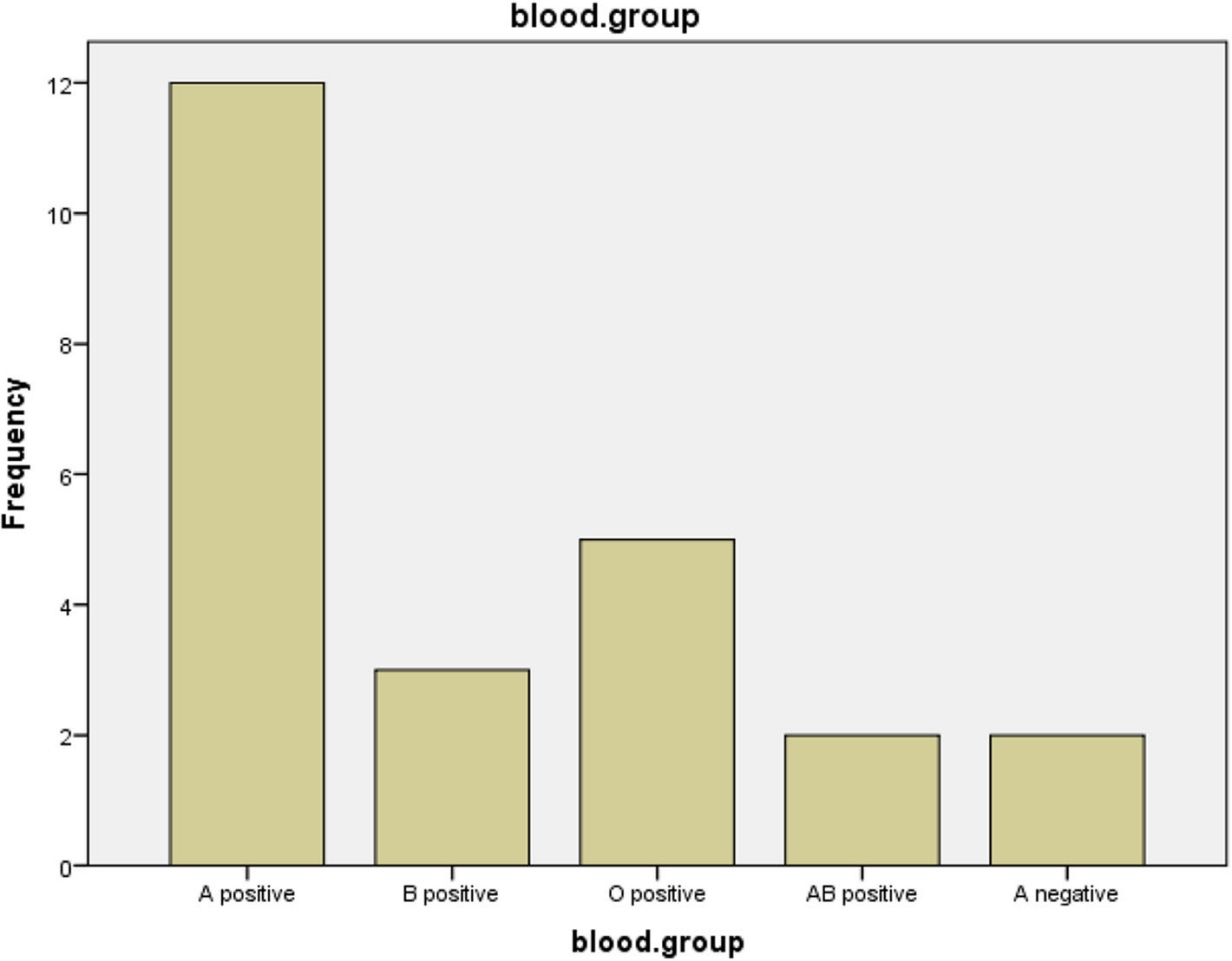
Frequency of blood groups

Most cases were type A blood group (58.3%) [12 cases were A^+^ (50%) and 2 were A^−^ (8.3%)], 5 cases were O^+^ (20.8%), and 3 cases were B^+^ (12.5%); there were no blood groups of B^−^, AB^−^, or O^−^ (Fig. 1). The SpO2 of 17 cases (70.8%) was in the normal range (over 93%), while 7 cases (29.2%) had lower SpO2.

### Laboratory results

The main laboratory changes are shown in Table 2. In all, almost half (45.8%) of cases showed lymphopenia. Of 13 normal cases, 3 cases had values between 1100 and 1200, and 4 cases had values between 1200 and 1300. The mean leucocyte count (WBC) was 10,380 (range: 3900–23,200) per microliter. One case had severe leukocytosis due to acute abdomen and bile vomiting (23200); by eliminating this case from the analysis, a mean WBC of 9800 (range: 3900–19,000) per microliter was the result. Considering pregnancy physiological leukocytosis, which makes up to 16,900 cells per microliter normal, only two cases had WBC of over 16,000. Of these two cases, one was a twin pregnancy (WBC = 19,000) which was referred at the 33th gestational week with the complaint of rupture of membranes and a decrease in the amniotic fluid index; in this case, a Caesarean section was performed on the fourth day of hospitalization, the patient developed fever, tachycardia, and cough on the day of the operation, and subsequent PCR test and CT scan were positive. The second case of leukocytosis was related to severe preeclampsia with HELLP syndrome.

**Table 2:** Laboratory characteristics of pregnant patients with COVID- 19

Characteristic	n/N (Percent)
Leucocytosis	2/24 (8.3)
Leucopenia	0
Lymphopenia	11/24 (45.8)
thrombocytopenia	0
High AST	2 /18 (11.1)
High ALT	3/18 (16.6)
High Creatinine	1/22 (4.5)
High LDH	1/19 (5.2)
Elevated C-reactive protein	18/22 (81)
Anemia	10/24 (41.6)

Regarding neutrophils percentage, the mean was 80% (range: 57–97%), in which fact the obvious increase in neutrophils is evident. The mean platelet count was 198,000 (range: 102000–333,000) per microliter, which was in the normal range. The mean of blood hemoglobin was 11.37 (range: 8.9–13.7) g/dl. Due to physiological anemia in pregnancy, the minimum normal values of the first, second, and third trimesters are considered to be 11, 10.5, and 11, respectively, considering gestational age. There were 10 anemic cases (41.6%); 1 in the first, 2 in the second and 7 in the third trimester, while 59% had normal hemoglobin. The minimum value was related to a 37-week pregnancy with underlying cardiac disease.

Liver enzymes (ALT and AST) were assessed in 18 cases; according to research, ALT ≥45 units per liter and AST ≥ 35 were considered abnormal. Of 18 cases, ALT was in the normal range in 16 cases (88.9%) and increased in 2 cases. AST was also normal in 15 cases (83.3%), and was increased in 3 cases (16.7%); a great increase was observed in 1 case (ALT = 122 and AST = 130), who was referred at the 36th week of gestation with fever, dry cough, and myalgia, and underwent labor induction and vaginal delivery due to fetal bradycardia; in this case, the CT scan was positive, but the PCR test was negative. LDH was assessed in 19 cases, with a mean of 439 (range: 265–645) units per liter (SD = 110.5). According to research, LDH in the third trimester is considered normal up to 524. However, LDH is an important value in determining the severity of preeclampsia; one of the high LDH cases in this study was due to HELLP syndrome (LDH = 645), and this case underwent emergency Caesarean section due to severe preeclampsia and placental abruption at the 28th week where the neonate ad expired due to severe preeclampsia, IUGR and chronic abruption. ESR was increased in only one case (ESR = 95 mm/hr), the case with HELLP syndrome. According to the literature, ESR may increase normally up to 70 in the third trimester. Creatinine was assessed in 22 cases, with a mean of 0.76 (range: 0.5–1) milligrams per deciliter (SD = 0.121). Compared to normal values of pregnancies, this value was increased in just 1 case. BUN was assessed in 16 cases, mean was 16.5 (range: 8–39) mg/dl, SD = 8.4) which was increased in 9 cases (56.3%). CRP was assessed in 22 cases, which was increased in 81.2% of cases. The blood pH of 6 neonates was assessed; except for 1 case related to HELLP syndrome, all were in the normal range.

### Pregnancy outcomes

A total of 12 cases were under ongoing normal pregnancy care. Of the 12 remaining cases, 10 cases delivered, 9 through Caesarean section and 1 vaginally. Two cases underwent therapeutic abortion, one for fetal anomaly, while the other case was hospitalized for pregnancy termination because of embryo death with underlying disease of mother (cirrhosis and colitis). This case developed dyspnea on the third day after admission; a PCR test was conducted and was positive.

One case was referred after a Caesarean section; the Caesarean indication was premature rupture of membranes and failure to induce labor. This case developed cough, fever, tachycardia, and saturation loss in the recovery room and was referred to Al-Zahra hospital. At the neonatal follow-up, the baby tested negative for PCR with a good health status.

The leading cause of Caesarean section in 4 cases was preeclampsia; 2 of them developed HELLP syndrome and eclampsia, with a fever developing after Caesarean in the case of the patient with HELLP syndrome, while the other case had a history of open heart surgery in childhood.

The case of twin pregnancy underwent Caesarean section due to rupture of membranes and decreased amniotic fluid index at 32–33 weeks of gestation. The mother developed fever, tachycardia and cough prior to surgery, a Caesarean was done under general anesthesia, the mother’s PCR test was positive after surgery, her SpO2 was 98% without oxygen therapy, and blood values were in normal ranges. The two preterm neonates were a girl and a boy who were admitted to the NICU due to subcostal retraction and were discharged with good general health status and negative PCR tests and were recommended to continue home quarantine. The general characteristics of mothers and neonates are shown in Table 3.

**Table 3:** Summary of maternal characteristics, symptoms and delivery details of pregnant women with SARSCov-2^a^ infection

Patient	Age	Blood group	Gestational age at diagnosis (weeks+ days)	Maternal comorbid conditions	Symptoms	Mode of delivery	Fetus Weight (grams)	Apgar score (1 min; 5 min)	Neonatal SARSCov-2 PCR^b^
1	29	O^+^	34	Severe preeclampsia	Dyspnea,	c/s ^c^	950	6/10;8/10	Negative
2	23	A^+^	36+ 5	None	Fever, dyspnea	c/s	3010	8/10;9/10	Negative
3	20	A^+^	39	None	Cough, tachypnea	c/s	3500	9/10;10/10	Negative
4	20	A^−^	33 (twins)	None	Fever, cough, tachycardia	c/s	1830 2030	6/10;8/10 8/10;9/10	Negative Negative
5	33	A^+^	31+ 4	Severe preeclampsia	Cough	c/s	1505	1/10;6/10	Negative
6	36	O^+^	28+ 1	HELLP syndrome	Fever	c/s	680	1/10;1/10	Negative
7	26	B^+^	36+ 6	Heart surgery	Cough+ myalgia	c/s	2470	9/10;10/10	Negative
8	37	O^+^	40+ 2	Severe preeclampsia	Cough	c/s	3750	9/10;10/10	Negative
9	22	B^+^	36+ 2	None	Cough+ myalgia	NVD ^d^	2800	9/10; 10/10	Negative
10	31	A^+^	31+ 2	Acute abdomen +GTT disorder	Fever, dyspnea,, low o_2_ sat	c/ s	2500	IUFD	Not assessed
11	25		35+ 3	None	Dry Cough, anosmia,	NVD	–	9/10;10/10	Negative
12	39		13+ 6	Cirrhosis + colitis	Dyspnea	Abortion	–	–	–
13	22		26	Hypothyroid	Fever, dry cough	c/ s	–	9/10;10/10	Negative
14	32		27	None	Fever, dry cough, dyspnea	c/s	–	9/10;10/10	Negative
15	22		13.4	None	Fever, dry cough	c/ s	–	9/10;10/10	Negative
16	30		21.2	None	Dry cough	c/s	–	9/10;10/10	Negative
17	28		29.4	None	Fever, dry cough, dyspnea, diarrhea, tachycardia	c/ s	–	9/10;10/10	Negative
18	24		14.2	None	Cough	c/ s	–	9/10;10/10	Negative
19	31		33	None	Fever, cough,	c/s	–	9/10;10/10	Negative
20	17		28.2	None	Myalgia, tachycardia	c/ s	–	9/10;10/10	Negative
21	24		26	Hypothyroid	Cough, fever,	NVD	–	IUFD due to hypertension	–
22	20		21.3	None	Dyspnea	c/ s	–	9/10;10/10	Negative
23	26		37.1	GTT disorder ^e^	Dyspnea	c/ s	–	9/10;10/10	Negative
24	18		13	Hypertension	Fever, dyspnea	Abortion	–	–	–

*Abbreviations*: ^a^ *SARS-Cov-2* severe acute respiratory syndrome coronavirus 2, ^b^ *PCR* polymerase chain reaction, ^c^ *C/S* cesarean section, ^d^ *NVD* Normal Vaginal Delivery, ^e^ *GTT* Glucose Tolerance Test

Another case was suspicious acute abdomen with bile vomiting at 31–32 weeks of gestation which led to sudden intrauterine fetal death (IUFD) few hours after laparotomy and underwent Caesarean for labor induction failure. Therefore, in all cases, performing Caesarean was under obstetric indication and was not associated with COVID-19 infection. Out of 8 Caesarean cases, three cases underwent general anesthesia and 5 underwent spinal anesthesia; general anesthesia was used for emergency delivery.

Regarding underlying disease, preeclampsia (in 4 cases), GTT disorder (in 2 cases), HTN (in 1 case), hypothyroidism (in 2 cases), history of open heart surgery in childhood (in 1 case), history of cirrhosis and colitis (in 1 case) and liver enzyme disorder (in 1 case) were observed.

Two cases with A^+^ blood group were admitted to the ICU, and 1 was referred with positive PCR, protracted diarrhea, hypokalemia, and severe dehydration, Spo2 = 85% and D-Dimer = 5603, at 29 weeks of gestation; she was discharged with general good health waiting for delivery at term. The second case was a nulliparous in the 31st week of gestation without underlying disease who was referred with a stomachache and protracted biliary vomiting, severe acidosis, and GTT disorder, and a positive CT scan, high leukocytosis (WBC = 23,200), lymphopenia (lymph = 5%), neutrophilia (Neut = 91%), normal hepatic and renal tests, fasting blood sugar no more than 160; however, the clinical symptoms of diabetic acidosis were manifested. The patient underwent exploratory laparatomy with diagnosis of acute abdomen and acidosis, in which no specific clinical findings were achieved; after laparatomy, the fetus developed sudden IUFD, and the mother underwent a Caesarean because of failed labor induction. The outcome was a 2500 g dead male; there were no symptoms of placental abruption. According to the national protocol, PCR tests and/or any assessment of COVID-19 infection are not done for expired cases, therefore, a PCR test was not done for the neonate, and no information was available on neonatal COVID-19 infection. The cause of clinical symptoms, IUFD, and its association with COVID-19 remains controversial.

### Neonatal outcomes

During admission 11 neonates were born through 10 deliveries (1 case was twins). In all, 20% of cases were term pregnancies and 80% were under 37 weeks, of which 50% were under 34 weeks, and the remaining were among 34–37 weeks.

Over 11 neonates there were 5 girls and 6 boys; PCRs of all live neonates were negative. Neonatal outcomes were good in 8 of 11 cases (72.7%) according to gestational age. Apgar scores were good except in 2 cases (severe IUGR at 28 weeks and preeclampsia at 31–32 weeks. Apgar scores and neonatal weights were affected by maternal underlying disease and gestational age. Neonatal weight of 2 cases were very low (680 and 950 g for 28 and 34 weeks, respectively); the total neonatal mean weight was not computable because of different birth ages.

There were 5 cases of NICU hospitalization, all of which were preterm (31 to 36 weeks and 5 days) and all of which were discharged with good general health. There were 2 mortality cases among 11 neonates. Blood pH was assessed in 6 neonates due to prematurity and NICU admission, which were in the normal range except for 1 case (related to HELLP syndrome). PCR tests were negative for all live neonates. At the follow up of the other 12 ongoing pregnancies, 11 delivered at term, 10 through Caesarean, mostly because of previous Caesarean section or maternal demand. One pregnancy ended in IUFD because of pregnancy hypertension and was induced for vaginal delivery. All live babies were healthy.

## Discussion

In the current study, the mean age of the mothers was 26.5 years and most were in the third trimester (62.5%), which was in line with previous studies [10, 12, 13]. It could be attributed to physiologic changes in pregnancy which include upper airway edema and hyperemia, decreased functional residual capacity, rapid oxygen consumption, pregnant uterine pressure, increased tidal volume and minute ventilation, and decreased lower esophageal sphincter tone, which increase mucosal fragility, decrease upper airway diameter, physiological respiratory alkalosis and increase the risk of aspiration [14].

In the current study, the most common symptoms of COVID-19 were fever and cough, which was in line with the results of the recent studies [11, 13, 15, 16]. Symptoms which are similar for non-pregnant women usually include fever, cough, myalgia, fatigue, dyspnea, sore throat, anosmia, and/or gastrointestinal symptoms such as diarrhea [11, 15]. However, studies have reported severe maternal pneumonia as asymptomatic; it suggests that considering clinical symptoms as the main determinant of infection prognosis may lead to high maternal-fetal morbidity. Therefore, to reduce morbidity, all affected pregnancies should be screened and monitored thoroughly [1, 4, 16, 17].

Based on our findings, 10 cases delivered during the admission period, 9 through Caesarean section and 1 vaginally. Later follow-up with the remaining (12) pregnancies who were discharged after remission found that of the 9 who could be located, 8 had delivered healthy term babies under obstetrics indications. There was no increase in preterm labor or Caesarean section due to COVID-19 complications; Caesarean sections, if they occurred, were obstetrically indicated. These findings were in line with most of studies [18–20], although Nikpour et al. and Mullins et al. found an increase in premature delivery and Caesarean section which was inconsistent with the current study [15, 21–24]. It is hypothesized that placental trophoblasts may be susceptible to infection by COVID-19 in early or mid-pregnancy but this may be lower at term as the result of differential expression of ACE2 throughout gestation [20].

The blood group of most cases was A (58.3%), which was in line with other studies. Potential clinical implications of this finding may be more surveillance and personal protection of blood type A people, especially in pregnancy [25].

Regarding laboratory changes, discriminating count of white blood cells hemoglobin, platelet, hepatic enzymes, creatinine, ESR, CRP, LDH, and BUN were assessed, which mostly were in normal ranges, except one case with a high increase in ESR and LDH in a HELLP syndrome related case. The most common laboratory results were lymphopenia (45%), neutrophilia (80%), and elevated CRP (81%) which was consistent with the results by Vakili et al., Nikpour et al. and most other studies [15, 19, 26]. Although physiological lymphopenia occurs during normal pregnancy, a sharp decline in lymphocytes is used as a consistent predictor of COVID-19-associated mortality [4].

According to the results of other studies, no increase in mortality was reported at the third trimester [4, 12], which is inconsistent with the results by Shojaei et al. [21], and there is need for more research in twin pregnancies. The difference in the mortality rate of twins may be due to the difference of chorionicity [21]. Regarding outcomes by method of anesthesia, no difference was observed, which was in line with the study by Rong et al. [27]. It is concluded that there is no preferred method for anesthesia of COVID-19 confirmed pregnant mothers during Caesarean. The method depends on the anesthesiologist’s decision considering the patient’s overall condition.

There were two cases of ICU admission which were both were discharged with good general health. Neonatal outcomes in 8 of 11 neonates were good (72.72%), and neonatal morbidity and mortality in most cases resulted from pregnancy complications; no cases of vertical transmission of COVID-19 were reported, which was in line with most studies [16, 26, 28]. However, in the assessment of neonatal antibodies by Hui et al., despite negativity of PCR for all neonates, 5 neonates who were isolated from mothers immediately after delivery showed high antibodies, which represents the probability of placental contamination during delivery. Although maternal antibodies transfer to the fetus late in the second trimester, vertical transmission during labor needs further investigation [10, 13, 29–36].

### Implications

Case series and case reports in this critical epidemic time are of great value. The findings of this study can be used in the evaluation and management of pregnant women with COVID-19. Most patients in this survey were in their third trimester; these findings can be used to compare clinical symptoms and laboratory results with patients in their first and second trimesters. At a time when high quality studies on COVID-19 in pregnancy have not yet been performed, case reports and case series provide valuable and necessary information to guide physicians in patient management. Considering clinical symptoms as the main determinant of infection prognosis may lead to high maternal- fetal morbidity. Therefore, all affected pregnancies should be screened and monitored thoroughly.

### Strengths & Limitations

The sample size was limited and there is a need for more studies in this field.

## Conclusions

Findings suggest that clinical symptoms in pregnancy are similar to non-pregnant women. According to maternal and neonatal outcomes, no rise in risk of maternal death, premature labor, abortion or type of delivery was seen. Vertical transmission was not observed in any of the cases. Lymphopenia was the leading laboratory change. Given asymptomatic cases, despite severe forms of infection in pregnancies, we propose screening in all suspected cases. All placentas and newborns should be tested in the field for vertical transmission.

## Data Availability

The datasets used and/or analyzed during the current study are available from the corresponding author upon reasonable request.

## Abbreviations

WBC: White Blood Cell
COVID-19: Coronavirus Disease 2019;
ESR: Erythrocyte Sedimentation Rate
SARS-CoV-2: Severe Acute Respiratory Syndrome coronavirus-2
AST: Aspartate amino Transferase
ALT: Alanine aminotransferase
LDH: Lactate Dehydrogenase
PCR: Polymerase Chain Reaction
CRP: C-reactive protein
BUN: Blood Urea Nitrogen
HELLP: Hemolysis, Elevated Liver Enzymes, Low Platelet
GTT: Glucose Tolerance Test
HTN: Hypertension
mg/dl: milligram per deciliter g/ dl gram per deciliter
